# Measuring What Matters in Long-Term Care: Common Data Elements in Brazil, China, and the United States

**DOI:** 10.1093/geroni/igab046.608

**Published:** 2021-12-17

**Authors:** Michael Lepore, Kirsten Corazzini, Sheryl Zimmerman

## Abstract

Internationally sharable common data elements on residential long-term care (LTC) settings, such as nursing homes and assisted living facilities, can facilitate comparisons across diverse LTC settings for valuable insights on LTC regulation and oversight, practice and operations, infrastructure development, human resources issues, and quality and safety. However, such insights are predicated on the premise that data elements capture information that matters to the full LTC community, including residents, relatives and staff, and are able to be collected across diverse care settings, including low-resource contexts. A critique of much current LTC measurement is its focus on deficits and loss, rather than thriving, person-centered care, and healthy aging, which have been established as important to LTC communities internationally. Further, measurement burden, cultural differences in perceptions of data sharing, and data infrastructure differences are key issues for international data. An international collaborative of LTC researchers—Worldwide Elements to Harmonize Research in Long-Term Care Living Environments (WE-THRIVE)—has developed a set of common data elements that are recommended for parsimoniously assessing key outcomes, workforce and staffing, person-centered care, and the contexts within which LTC settings operate. The studies in this symposium provide insights into the validation and implementation of WE-THRIVE recommended measures in diverse, low-resource LTC contexts, including LTC settings in Brazil, China, and rural Midwest US. Study findings validate WE-THRIVE measures, and provide new knowledge to inform capacity-building for the measurement of person-centered care and healthy aging outcomes in diverse, low-resource, LTC settings.

